# Effects of imatinib mesylate (STI571, Glivec) on the pharmacokinetics of simvastatin, a cytochrome *P*450 3A4 substrate, in patients with chronic myeloid leukaemia

**DOI:** 10.1038/sj.bjc.6601152

**Published:** 2003-11-11

**Authors:** S G O'Brien, P Meinhardt, E Bond, J Beck, B Peng, C Dutreix, G Mehring, S Milosavljev, C Huber, R Capdeville, T Fischer

**Affiliations:** 1 Department of Haematology, University of Newcastle Medical School, Royal Victoria Infirmary, New Castle NE1 4LP, UK; 2 III. Med. Klinik und Poliklinik, Langenbeckstr, Mainz 155101, Germany; 3 Novartis Pharma AG, Basel, Switzerland; 4 Novartis Pharmaceuticals, East Hanover, NJ, USA

**Keywords:** imatinib, Glivec, Gleevec, CML, simvastatin, pharmacokinetics, cytochrome *P*450, CYP3A4

## Abstract

The inhibition by imatinib of the cytochrome *P*450 3A4 isoenzyme may reduce the CYP3A4-mediated metabolic clearance of clinically important coadministered drugs. The main purpose of this study was to evaluate the effect of the coadministration of imatinib on the pharmacokinetics of simvastatin, a probe CYP3A4 substrate. In total, 20 patients with chronic myeloid leukaemia received an oral dose of 40 mg of simvastatin on study day 1. On study days 2–7, each patient received 400 mg of imatinib once daily orally and on study day 8, 400 mg imatinib together with 40 mg of simvastatin was given. Blood levels of simvastatin were measured predose and for 24 h postdose on study days 1 and 8. Two additional blood samples were taken for imatinib pharmacokinetic (PK) assessment on day 8 before, and 24 h after, imatinib administration. Imatinib increased the mean maximum concentration (*C*_max_) value of simvastatin two-fold and the area under concentration–time curve (AUC _(0–inf)_) value 3.5-fold (*P*<0.001) compared with simvastatin alone. There was a statistically significant decrease in total-body clearance of drug from the plasma (CL/F) with a mean reduction of 70% for simvastatin (*P*<0.001): the mean half-life of simvastatin was prolonged from 1.4–2.7 h when given together with imatinib. No changes in imatinib PK parameters were found when given concomitantly with simvastatin. In conclusion, the coadministration of imatinib at steady state with 40 mg simvastatin increases the exposure (*C*_max_ and AUCs) of simvastatin significantly (*P*<0.001) by two-three-fold. Caution is therefore required when administering imatinib with CYP3A4 substrates with a narrow therapeutic window. The coadministration of simvastatin with imatinib (400 mg) was well tolerated and no major safety findings were reported in this study.

Imatinib mesylate (STI571, Glivec®, Gleevec® – Novartis, Basel, Switzerland) is a potent competitive inhibitor of the tyrosine kinases associated with ABL (Buchdunger *et al*, 1996; Druker *et al*, 1996), KIT (Buchdunger *et al*, 2000; Heinrich *et al*, 2000), PDGFr (Buchdunger *et al*, 1996, 2000) and ARG (Okuda *et al*, 2001), which impedes the interaction of ATP with the SH1 domain of these proteins (Schindler *et al*, 2000), thereby inhibiting the phosphorylation of downstream target proteins. Imatinib is a phenylaminopyrimidine derivative and represents the first of a new class of drugs known as signal transduction inhibitors. Following initial phase I/II dose-escalation studies with imatinib (Druker *et al*, 2001a, 2001b), subsequent studies have demonstrated remarkable efficacy with minimal side effects mostly in Philadelphia-positive leukaemias (Kantarjian *et al*, 2002; Ottmann *et al*, 2002; Sawyers *et al*, 2002; Talpaz *et al*, 2002; O'Brien *et al*, 2003) but also in solid tumours (Joensuu *et al*, 2001). Currently, clinical trials using imatinib with and without concomitant chemotherapy are being conducted in a number of c-kit and PDGF-R-positive malignancies(Apperley *et al*, 2001; Fischer *et al*, 2001; Johnson *et al*, 2002).

Imatinib is a competitive inhibitor of CYP3A4, CYP2D6 cytochrome *P*450 isoenzymes as well as CYP2C9, CYP3A5 and CYP4A to a lesser extent (Novartis, unpublished data). Coadministration of inhibitors of CYP3A4 with drugs known to be substrates of this enzyme could potentially affect the pharmacokinetic parameters of the substrate drug, and could be also responsible for considerably increasing its side effects (Desager and Horsmans, 1996). Simvastatin, an inhibitor of 3-hydroxy-3-methylglutaryl-coenzyme A (HMG-CoA) reductase, is used as a lipid-lowering agent. It is uniquely metabolised by CYP3A4, is well tolerated and is therefore commonly recommended as the model drug for testing drug interactions involving CYP3A4 substrates (US Department of Health and Human Services, 1999). Drug interactions between CYP3A4 substrates such as simvastatin and CYP3A4 inhibitors are potentially clinically important and have been reported to potentially enhance the risk of myopathy and rhabdomyolysis (Walker, 1989; Todd and Goa, 1990; Berland *et al*, 1991; Smith *et al*, 1991; Garnett, 1995; Meier *et al*, 1995; Goodman & Gilman's, The Pharmacological Basis of Therapeutics, 2000). Accordingly, the prescription instructions for HMG-CoA reductase inhibitors often suggest caution regarding the potential of occurrence of drug interactions with substrates, inhibitors and inducers of CYP3A compounds (Walker, 1989).

We therefore hypothesised that coadministration of imatinib, as an inhibitor of the microsomal CYP3A4 enzyme system, could affect the elimination rate of simvastatin. The present study was undertaken to assess this potential pharmacokinetic interaction by evaluation of the simvastatin plasma concentration *vs* time profiles after coadministration with imatinib (US Department of Health and Human Services, 1999) and its effects on safety and tolerability in patients with chronic myeloid leukaemia (CML).

## MATERIALS AND METHODS

### Patient population

In an open-label, nonrandomised, one-sequence study, 20 adult patients with CML who were haematologically or cytogenetically resistant or refractory to interferon-*α*, or intolerant of interferon-*α* were enrolled. There were 10 male and 10 female patients the mean age (±s.d.) was 50.5 years (±13.4 years), weight ranged from 53 to 111 kg, and height from 158 to 192 cm. None of the patients had any past or present medical conditions that could affect the study results. Each patient gave written informed consent before taking part of the study, which was approved by the ethics committee of the Landesärztekammer Rheinland-Pfalz, Mainz (Germany) and of the University of Newcastle/Royal Victoria Infirmary (UK). The study was conducted in agreement with the declaration of Helsinki, as amended in Tokyo, Venice, Hong-Kong and Somerset West. Drug interaction studies performed in healthy volunteers commonly use a crossover design. However, one of the requirements of the present study was to test interactions with simvastatin at steady-state levels of imatinib. As it usually requires approximately 7 days to reach serum steady-state levels of imatinib, it was considered unethical to perform the study in healthy volunteers and therefore the study was conducted in patients with CML. As a corollary of this restriction, practical and ethical issues (e.g., wash-out phase) prevented the study being performed as a crossover design. The use of concomitant medications that could potentially alter the integrity of the PK analysis (e.g., altered absorption, distribution) was forbidden. The patients were asked to refrain from strenuous physical exercise (e.g., weight training, aerobics, football) for 7 days before dosing until after the study completion evaluation, from alcohol for 72 h before dosing until after the study completion evaluation and from intake of xanthine (e.g., caffeine) or grapefruit (known as a CYP3A4 inhibitor (Schmiedlin-Ren *et al*, 1997; Lilja *et al*, 1998; Kane and Lipsky, 2000))-containing food or beverages 48 h before dosing and during the whole study.

### Study design

On study days 1 and 8, patients reported to the study site around 1 h prior to dosing for baseline evaluations and were kept at the centre until 12 h postdosing (Table 1

**Table 1:** Blood sampling schedule for pharmacokinetic analyses

**Pretreatment period**	**Pretreatment period**	**Treatment period**	**Treatment period**	**Treatment period**	**Post-treatment**
Days −21 to −2: screening evaluations	Period I:	Period I:	Period II:	Period III:	End of study evaluations 24 h after dosing day 8
	Day −1: baseline evaluations	Day 1: 40 mg simvastatin	Days 2–7: 400 mg Glivec od	Day 8: 400 mg Glivec od+40 mg simvastatin	
		PK sampling		PK sampling	

). At 24 h after dosing, the patients reported again to the study site for the 24 h blood sampling (study days 2 and 8) and study completion evaluations (study day 9). Blood samples for determination of simvastatin plasma concentrations were taken up to 24 h after dosing on study days 1 and 8. Imatinib was administered daily starting at day 2 at a dose of 400 mg (supplied as 100 mg hard gelatine capsules). Simvastatin (40 mg tablets of Denan® – Boehringer Ingelheim) for both centres was purchased by the pharmacist of the University of Mainz's Hospital. On study days 1 and 8, 40 mg of oral simvastatin was administered immediately after a low fat breakfast (on day 8, simvastatin and imatinib were given at the same time). No fluid intake apart from the fluid given at the time of drug intake was allowed until 2 h after dosing. Owing to the inherent risk of either reduced activity or enhanced toxicity of the concomitant medication and/or imatinib, drugs known to be metabolised by the same CYP450 isoenzymes as imatinib, were forbidden. Allopurinol 300 mg daily (an inhibitor of CYP2C9/10 (Yokochi *et al*, 1982; Veronese *et al*, 1991; Kane and Lipsky, 2000)) was recommended for patients with WBC 20.0 × 10^9^ l^−1^. Following the PK study, patients continued with therapeutic imatinib at the standard dose.

### Blood sampling

All blood samples were taken by either direct venepuncture or an indwelling cannula inserted in a forearm vein at predose (0 h), 0.5, 1, 2, 3, 4, 6, 10, 12 and 24 h after dosing on days 1 and 8 (Table 1). Immediately after the blood was drawn, each tube was inverted gently several times to ensure the mixing of tube contents (e.g., anticoagulant) and prolonged sample contact with the rubber stopper was avoided. The upright tube was kept on ice, and within 30 min the sample was centrifuged at 3 and 5°C for 10 min at approximately 1500 **g**. Immediately after centrifugation, at least 2 ml plasma was transferred to a polypropylene screw-cap tube put on dry ice. The tubes were kept frozen at ⩽−18°C pending analysis.

### Drug analysis

Simvastatin and simvastatin hydroxy acid with lovastatin as internal standard were determined in plasma by LC/MS/MS. The LC/MS/MS analyses were carried out on a Sciex API3000 mass spectrometer. The instrument was operated in the ESI mode (positive ion for drug, negative ion for metabolite) with selected reaction monitoring. LC was performed on a Shimadzu LC system operated in isocratic mode with a 2.0 × 50 mm^2^ C-18 column. Samples were prepared using a solid-phase extraction procedure. All concentrations are reported in terms of the free acid form of simvastatin and simvastatin hydroxy acid.

### Data analysis

All completed patients were included in the pharmacokinetic data analysis. For plasma concentrations of simvastatin the following parameters were determined: AUC_(0–*t*)_ (area under the concentration–time curve from time zero to *t*), AUC_(0–*∞*)_ (area under the concentration–time curve from time zero to infinity), *C*_max_ (maximum plasma drug concentration), *t*_max_ (time to reach maximum concentration following drug administration), *t*_1/2_ (elimination half-life associated with terminal slope of a semilogarithmic concentration–time curve), *V*_*z*_/*f* (apparent volume of distribution based on terminal phase of plasma concentration–time curves) and CL/F (total body clearance of drug from the plasma), in order to assess the effects of imatinib on the PK of simvastatin.

### Statistical analysis

The following pharmacokinetic parameters were used to assess an interaction of imatinib on simvastatin: AUC_inf_, AUC_all_, *C*_max,_ *V*_*z*_/*f*, CL/f, *t*_1/2_ and *t*_max_. With the exception of *t*_1/2_ and *t*_max_, parameters were ln-transformed prior to analysis. Treatment differences were assessed by *t*-tests. The means of differences of ln-transformed data together with 90% confidence intervals were then antilogged in order to get confidence intervals for the ratio ‘simvastatin+imatinib/simvastatin’. An interaction of imatinib with simvastatin was assumed, if these confidence intervals were not included in the ‘no-effect’ interval (0.80, 1.25). *T*_max_ was analysed nonparametrically. The alpha-level was set to 0.05 and no alpha-adjustment was made for multiple testing.

## RESULTS

### Drug safety and tolerability

In total, 14 (70%) of the 20 recruited patients reported a total of 30 adverse events. All but one adverse events were rated by the investigators as mild (grade 1) to moderate (grade 2). Of these, 12 patients had at least one adverse event grade 1, and four patients at least grade 2. Only one patient experienced a grade 3 left leg cellulitis, but this was assessed as not related to the study drugs. No deaths occurred during the course of the study and none of the adverse events resulted in discontinuation from the study. The most common adverse events reported were neurological symptoms (headache, insomnia), gastrointestinal symptoms (nausea, loose stool), and musculoskeletal symptoms (myalgia, muscle cramps cramping, pain in limb).

### Pharmacokinetics of simvastatin

The main pharmacokinetic parameters of simvastatin and its hydroxy acid metabolite, for the 20 CML patients determined by noncompartmental model analyses, are listed in Tables 2

**Table 2:** Simvastatin PK parameters following oral administration of 40 mg simvastatin alone and combined with oral administration of 400 mg imatinib

	**Simvastatin plus imatinib**	**Simvastatin alone**
*t*_max_ (h)^a^	1.0 (0.5–3.0)	1.0 (0.5–4.0)
*C*_max_ (ng ml^−1^)	42.3±25.8	23.3±23.8
*t*_1/2_	2.7±1.3	1.4±0.8
AUC_(0–all)_ (ng h ml^−1^)	136.4±113.6	45.5±61.1
AUC_(0–*∞*)_ (ng h ml^−1^)	137.7±110.2	47.2±60.4
*V*_*z*_/*F*(l)	1543.0±810.9	3115.9±2749.9
CL/F (l h^−1^)	504.1±431.8	2000.3±1975.3

a Median (range). All unflagged values are mean±s.d. Mean and s.d. values are shown.

and 3

**Table 3:** PK parameters of simvastatin hydroxy acid following oral administration of 40 mg simvastatin alone and combined with oral administration of 400 mg imatinib

	**Simvastatin plus imatinib**	**Simvastatin alone**
*t*_max_ (h)^a^	1.0 (0.5–3.0)	1.0 (0.5–4.0)
*C*_max_ (ng ml^−1^)	24.9±19.3	14.5±13.3
*t*_1/2_	3.3±1.4	2.4±1.1
AUC_(0–all)_ (ng h ml^−1^)	116.1±104.2	44.3±41.7
AUC_(0–*∞*)_ (ng h ml^−1^)	119.9±106.0	51.9±39.9

Mean and s.d. values are shown. See Table 2.

**Figure 1:**
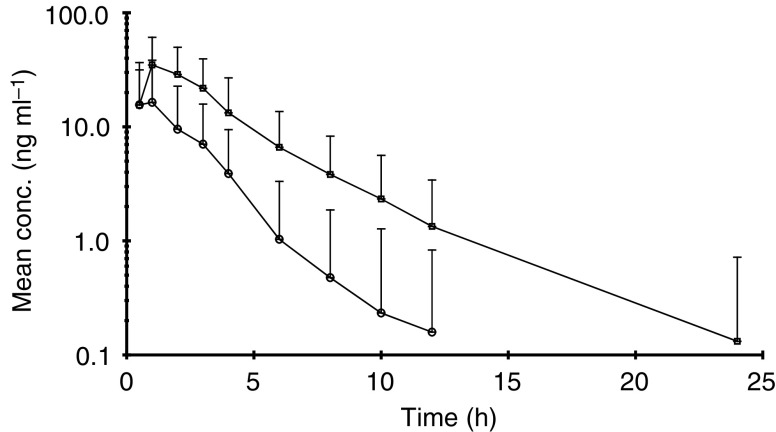
Plasma concentrations (mean+s.d.) of simvastatin following oral administration of simvastatin alone (○) and combined with imatinib (□).

. The mean and standard deviation for each parameter are given for the two treatment periods in which simvastatin was administered. Figures 1

and 2

**Figure 2:**
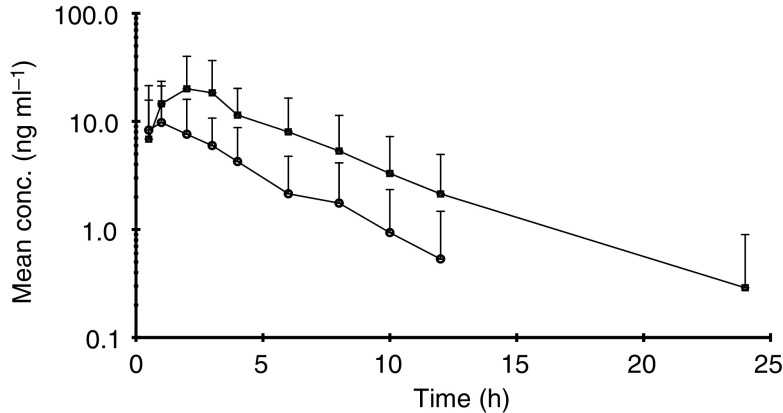
Plasma concentrations (mean+s.d.) of simvastatin hydroxy acid following oral administration of simvastatin alone (•) and combined with imatinib (▪).

show the mean plasma concentrations of simvastatin and its metabolite (simvastatin hydroxy acid), respectively, following either oral administration of simvastatin alone or combined with oral administration of imatinib. Figure 3

**Figure 3:**
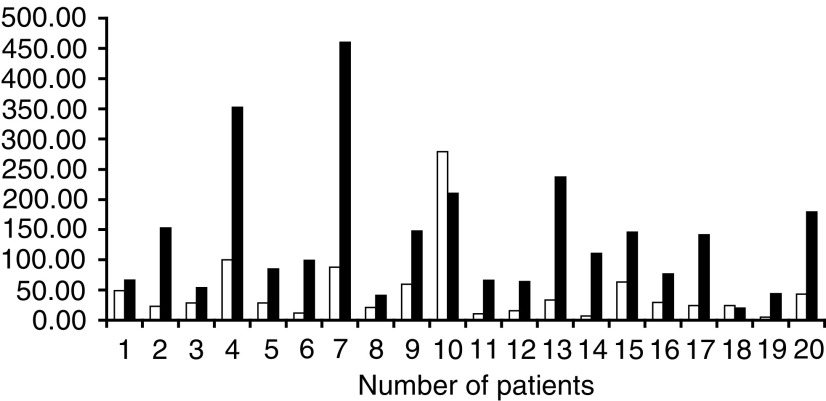
Comparison of AUC_(0–*∞*)_ of simvastatin following oral administration of simvastatin alone and combined with Glivec® (STI571). Simvastatin alone shown in open columns, simvastatin plus imatinib shown in black columns.

shows the comparison of plasma AUC_(0–*∞*)_ of simvastatin following oral administration of simvastatin alone and combined with oral administration of imatinib in 20 subjects. Following imatinib coadministration, the mean simvastatin *C*_max_, AUC_(0–all)_ and AUC_(0–*∞*)_ increased significantly by two-to-three-fold (*P*<0.001). There was a statistically significant decrease in CL/F with a mean reduction of 70% (*P*<0.001). With regard to metabolites, the mean *C*_max_ and AUCs of simvastatin hydroxy acid also increased significantly by two-to-three-fold (*P*<0.001) after imatinib treatment (Table 3 and Figure 2). The coefficient of variation (CV) for *C*_max_ and AUCs showed considerable interpatient variation. The mechanism for this variability is not clear yet but could be attributed to interpatient variations in CYP3A4 activity. Compliance to imatinib treatment and plasma concentration at steady state were checked by the analysis of the plasma samples taken on study days 8 and 9 in the morning prior to administration. The mean plasma imatinib trough concentrations were similar on day 8 (1268 ng ml^−1^) and day 9 (1182 ng ml^−1^) indicating that PK steady state for imatinib was reached in those patients after 6-day oral doses.

## DISCUSSION

This study was performed to determine whether imatinib could alter the pharmacokinetics of a single dose of simvastatin when given concomitantly in patients with chronic myeloid leukaemia. This has important implications because of potential interactions of imatinib with commonly prescribed drugs in the clinic.

The major route of degradation of simvastatin within the body is by cytochrome *P*450 3A4-mediated biotransformation (Vickers *et al*, 1990; Prueksaritanont *et al*, 1997) although the drug can be converted reversibly to simvastatin hydroxy acid by esterases. From *in vitro* drug interaction studies, CYP3A4 was also found to be the major human *P*450 enzyme involved in the microsomal biotransformation of imatinib (data not shown). Simvastatin inhibits HMG-CoA reductase causing decreases in intrahepatic cholesterol and upregulation of LDL-receptors with enhanced clearance of LDL and other apolipoprotein B containing lipoproteins from the circulation. It appears that these interactions do not have a relevant clinical effect on the efficacy of the HMG-CoA reductase inhibitors to reduce the cLDL from the plasma, but the concomitant administration of HMG-CoA reductase inhibitors and cyclosporine, fibrate or nicotinic acid may enhance the risk of myopathy or rhabdomyolysis (Berland *et al*, 1991; Smith *et al*, 1991; Meier *et al*, 1995).

The coadministration of simvastatin with imatinib (400 mg) was well-tolerated and no major safety concerns were reported in this study. No clinically significant abnormalities in laboratory values, vital signs or ECGs were reported. The majority of the adverse events were assessed as grade 1/2 and no myopathy or rhabdomyolysis occurred. Only one grade 3 left leg cellulitis (which required hospitalisation) was reported but was not related to the study drugs. This study shows that coadministration of imatinib increased the mean *C*_max_ value of simvastatin two-fold and the AUC_(0–*∞*)_ value three-fold compared with simvastatin alone and the mean half-life of simvastatin was prolonged from 1.4 to 2.7 h when given together with imatinib. This indicates an inhibition of CYP3A4 by which the oxidative biotransformation of simvastatin to other metabolites is primarily mediated. It was also observed that the formation of simvastatin hydroxy acid from simvastatin by esterases is not prevented by imatinib, which explains the increases in both simvastatin and simvastatin hydroxy acid concentrations. This would suggest that in the presence of imatinib, plasma levels of standard doses of drugs which are degraded by the CYP3A4 system (Table 4

**Table 4:** Substrates of cytochrome *P*450 enzymes CYP3A3/4

**Substrates: imatinib may increase the potency of these drugs**
Acetaminophen, Alfentanil, Alosetron, Alprazolam, Amiodarone, Amitriptyline (minor), Amlodipine, Anastrozole, Androsterone, Antipyrine, Astemizole, Atorvastatin, Benzphetamine, Bepridil, Bexarotene, Bromazepam, Bromocriptine, Budesonide, Bupropion (minor), Buspirone, Busulphan, Caffeine, Cannabinoids, Carbamazepine, Cerivastatin, Cevimeline, Chlorpromazine, Cimetidine, Cisapride, Citalopram, Clarithromycin, Clindamycin, Clomipramine, Clonazepam, Clozapine, Cocaine, Codeine (demethylation), Cortisol, Cortisone, Cyclobenzaprine (demethylation), Cyclophosphamide, Cyclosporine, Dapsone, Dehydroepiandrostendione, Delavirdine, Desmethyldiazepam, Dexamethasone, Dextromethorphan (minor, N-demethylation), Diazepam (minor;hydroxylation, N-demethylation), Digitoxin, Diltiazem, Disopyramide, Docetaxel, Dofetilide (minor), Dolasetron, Donepezil, Doxorubicin, Doxycycline, Dronabinol, Enalapril, Erythromycin, Estradiol, Ethinyl Estradiol, Ethosuximide, Etoposide, Exemestene, Felodipine, Fentanyl, Fexotenadine, Finasteride, Fluoxetine, Flutamide, Glyburide, Granisetron, Halofantrine, Hydrocortisone, Hydroxyarginine, Ifosfamide, Imipramine, Indinavir, Isradipine, Itraconazole, Ketoconazole, Lansoprazole (minor), Letrozole, Levobupivicaine, Lidocaine, Loratadine, Losartan, Lovastatin, Methadone, Mibefradil, Miconazole, Midazolam, Mifepristone, Mirtazapine (N-demethylation), Montelukast, Navelbine, Nefazodone, Nelfinavir, Nevirapine, Nicardipine, Nifedipine, Niludipine, Nimodipine, Nisoldipine, Nitrendipine, Omeprazole (sulfonation), Ondansetron, Oral contraceptives, Orphenadrine, Paclitaxel, Pantoprazole, Pimozide, Pioglitazone, Pravastatin, Prednisone, Progesterone, Proguanil, Propafenone, Quercetin, Quetiapine, Quinidine, Quinine, Repaglinide, Retinoic acid, Rifampin, Risperidone, Ritonavir, Salmeterol, Saquinavir, Sertindole, Sertraline, Sibutramine, Sildenalfil citrate, **Simvastatin**, Sirolimus, Sufentanil, Tacrolimus, Tamoxifen, Temazepam, Teniposide, Terfenadine, Testosterone, Tetrahydrocannabinol, Theophylline, Tiagabine, Tolterodine, Toremifene, Trazodone, Tretinoin, Triazolam, Troglitazone, Troleandomycin, Venlafaxine (N-demethylation), Verapamil, Vinblastine, Vincristine, Warfarin (R-warfarin), Yohimbine, Zaleplon (minor pathway), Zatoestron, Zileuton, Ziprasidone, Zolpidem, Zonisamide.

Simvastatin in bold is a substrate for CYP3A4 and in this study, coadministration with imatinib resulted in significantly increased mean maximum concentration and area under concentration–time curve values for simvastatin. One might expect coadministration with imatinib to increase the potency of these drugs. Caution is also required with drugs which induce or inhibit CPY3A4. Adapted from Cytochrome *P*-450 Enzymes and Drug metabolism. (In: Lacy CF, Armstrong LL, Goldman MP, Lance LL (eds) *Drug Information Handbook*, 8th edn. Hudson, OH: LexiComp Inc. 2000: pp 1364–1371).

and see also: http://medicine.iupui.edu/floc khart/) may be increased. For example, one might predict that the effects of warfarin, digoxin, certain antihypertensive agents (e.g., diltiazem, nifedipine, verapamil), steroids, benzodiazepines and other drugs commonly used in the practice of haematology (e.g., busulphan, cyclosporine, cyclophosphamide, doxorubicin etoposide, vincristine) could be enhanced and appropriate vigilance to avoid undesirable effects should be exercised. In addition, concomitant use of simvastatin or other HMG-CoA reductase inhibitors with imatinib may increase the risk of myopathy or rhabdomyolysis and again caution is required.

The design of the study allows only limited interpretation of the effects of simvastatin on plasma levels, and perhaps therefore efficacy, of imatinib. However there were no apparent effects of simvastatin on the PK of imatinib in these 20 patients, although more detailed PK studies would be required to resolve this issue definitively. Although this study was not designed to assess the potential relationships between the efficacy of imatinib and pharmacokinetics parameters, this important question is being addressed by ongoing population PK/PD modelling analyses within the context of ongoing phase II and phase III studies.

In conclusion, the coadministration of imatinib (400 mg) at steady state with 40 mg simvastatin significantly (*P*<0.001) increases the exposure (*C*_max_ and AUCs) to simvastatin by two-to-three-fold. This effect is most likely the result of the inhibition of CYP3A4-mediated metabolism of simvastatin in the liver and has implications for the monitoring of concomitant therapies in patients being treated with imatinib. Caution is therefore required when administering imatinib with CYP3A4 substrates with a narrow therapeutic window.
